# Effects of Psychosocial and Ergonomic Risk Perceptions in the Hospital Environment on Employee Health, Job Performance, and Absenteeism

**DOI:** 10.3390/healthcare13091000

**Published:** 2025-04-26

**Authors:** Kadriye Sönmez, Salim Yilmaz, Derya Karabay

**Affiliations:** 1 Department of Medical Documentation and Secretariat, Plato Vocational School, Istanbul Topkapi University, Istanbul 34087, Türkiye; kadriyesonmez@topkapi.edu.tr; 2 Department of Health Management, Faculty of Health Sciences, Acibadem Mehmet Ali Aydinlar University, Istanbul 34752, Türkiye; 3 Department of Health Management, Graduate School of Business, Sakarya University, Istanbul 54187, Türkiye; deryakarabay@arel.edu.tr

**Keywords:** hospital workers, psychological safety, ergonomic conditions, job performance

## Abstract

**Background:** This study examined the effects of psychosocial and ergonomic risk perceptions in the hospital environment on employee health, job performance, and absenteeism. In fast-paced hospital settings, the cumulative physical and psychological demands of patient care, exacerbated by poor ergonomic conditions, can jeopardize employee well-being and compromise service quality. **Methods:** A cross-sectional study was conducted among healthcare professionals in Istanbul using a multimethod approach with a quantitative emphasis. To analyze the interrelationships among these variables while controlling for demographic factors, including age and sex, structural equation modeling was employed. **Results:** The findings indicated that both psychological safety and favorable ergonomic conditions significantly enhanced job satisfaction, which in turn positively influenced mental health and overall job performance. Moreover, better physical health was associated with reduced absenteeism, further contributing to improved job performance. These results highlight the significance of developing supportive and ergonomically sound work environments for enhancing employee well-being, reducing absenteeism, and optimizing performance in hospital settings. **Conclusions:** This study offers valuable insights for hospital administrators and policymakers seeking to implement effective interventions that address psychosocial and ergonomic challenges, thereby ensuring higher quality healthcare services.

## 1. Introduction

In hospital settings, the physical and psychological demands of patient care compromise both employee well-being and service quality. Long working hours, inadequate equipment, and communication breakdowns contribute to burnout, job dissatisfaction, and absenteeism. These factors also undermine psychological safety, which is critical for performance and collaboration in healthcare teams [1,2,3,4,5]. Although studies on the long-term effects remain limited, employees experience burnout and other mental fatigue risks. More than 50% of nurses report working in high-stress environments [2]. Moreover, when these risks are disregarded, the margin for error increases, unscheduled absenteeism rates that disrupt workflow rise, and even risks such as violence against healthcare professionals are indirectly intensified. This condition fosters an environment where the sense of psychological safety deteriorates [5]. High absenteeism rates among healthcare professionals, particularly in low- and middle-income developing countries, support this argument [6]. Additionally, health issues, ranging from chronic pain to postural disorders, arise owing to the lack of ergonomic arrangements, reducing employee motivation and productivity. A previous study conducted in public hospitals highlighted how poor ergonomics heightened organizational conflicts and emphasized the significance of addressing psychosocial risks [7]. However, literature on this subject remains lacking. Nevertheless, it is well-documented and widely acknowledged that ergonomic issues present serious health concerns [8,9]. Studies have indicated that healthcare workers operating in environments with poor ergonomic conditions are more likely to experience musculoskeletal disorders [10].

When psychological safety is inadequately ensured, organizational learning is disrupted, and job performance significantly declines. Because 75% of workplace violence incidents occur in healthcare settings, low psychological safety perceptions further contribute to employees’ reluctance to freely express their opinions [11,12]. Workplace issues, including mobbing, incivility, and ostracism, damage employees’ sense of psychological safety [13,14]. Psychological safety, therefore, directly enhances job satisfaction by fostering an environment where employees can freely express themselves without fear of committing mistakes [15]. Research has shown that a workplace silence culture poses a threat to employees’ emotional well-being [1,16]. All these factors foster considerable psychosocial and ergonomic risks in hospital settings, resulting in widespread consequences affecting both employee health and healthcare service quality [8,17].

A critical factor that directly influences job performance among healthcare workers is job satisfaction. Higher job satisfaction enhances employee motivation, improving both patient care quality and overall productivity [3,18]. Conversely, low job satisfaction increases the risk of errors, fosters intentions to leave the profession, and threatens healthcare service sustainability [19,20]. In some countries, turnover intentions have reached 41%, reducing healthcare quality while positively correlating with absenteeism risks [20]. A study conducted in Australia and Iceland reported that 43% of newly hired nurses changed jobs within 12 months, and absenteeism rates among them ranged between 73% and 74% [20,21]. An empirical study investigating the high absenteeism rates in the healthcare sector revealed that managers recognize that this issue is linked to the inherent physical and mental health risks of the profession [22].

Although several studies have examined the impact of psychosocial and ergonomic factors on job satisfaction, physical and mental health, and absenteeism among healthcare workers, there is a lack of comprehensive research analyzing these multidimensional interactions within a single model. These relationships can cumulatively increase risks, including workplace accidents [4]. In fast-paced and complex institutions, such as hospitals, gaining a deeper understanding of these variables is strategically vital for both employee well-being and overall organizational performance [23]. Furthermore, addressing these concerns can increase the interest in healthcare professions, supporting the expansion of the healthcare workforce [24].

Unlike previous studies, this research offers a unified structural model that concurrently examines psychosocial and ergonomic risk perceptions and their effects on job satisfaction, mental and physical health, and absenteeism. By applying this model to a balanced sample from both public and private hospitals, this study provides a nuanced understanding of how institutional contexts shape employee performance. In addition, by including control variables such as sex and age, the analysis aims to isolate the independent effects of the core factors and enhance the validity of the findings.

## 2. Materials and Methods

This cross-sectional study was conducted from 15 January 2025 to 5 February 2025, during which data collection and analysis were performed to examine the relationships among job performance, psychological safety, ergonomic conditions, job satisfaction, mental and physical health, and absenteeism among hospital-based healthcare professionals.

**Figure 1:**
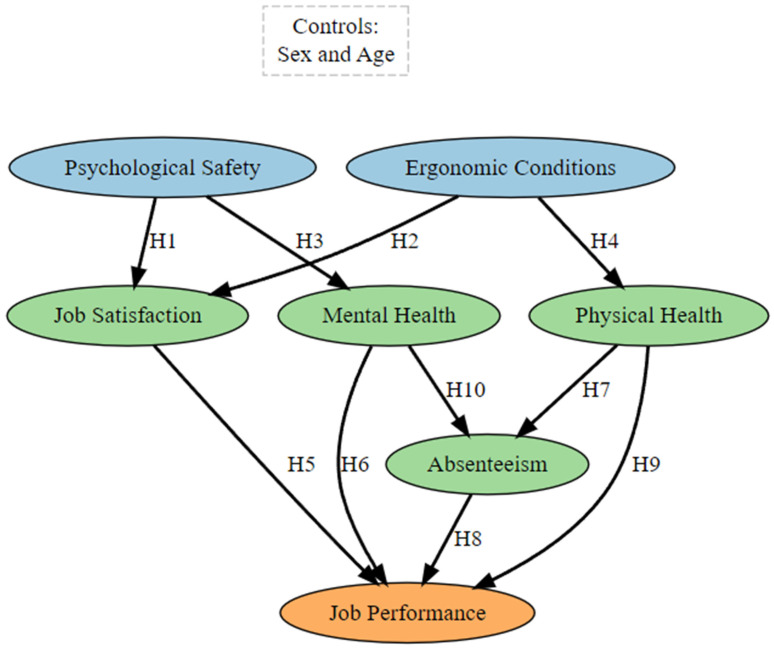
Theoretical model.

The theoretical model for this study, which examines the factors influencing job performance among hospital employees, is illustrated in Figure 1. The orange variable, job performance, serves as the dependent variable, whereas the green variables—job satisfaction, mental health, physical health, and absenteeism—are endogenous variables influenced by other factors in the model. The blue variables, psychological safety and ergonomic conditions, are exogenous variables directly affecting the endogenous variables but are not influenced by others in the model. The model outlines the hypothesized relationships (H_1_–H_10_) between these variables, with sex and age included as control variables to account for demographic effects. We believe that this framework offers a structured approach to understanding the dynamics influencing job performance in healthcare settings.

### 2.1. Theoretical Background

This theoretical model was designed to explore the intricate relationships affecting job performance among hospital employees, focusing on workplace conditions, individual well-being, and demographic factors. Psychological safety was included as it fosters a secure environment for open communication and trust, which are crucial for job satisfaction and mental health [25]. Ergonomic conditions were integrated owing to their role in improving physical and mental health by minimizing physical strain and promoting workplace comfort [26]. Job satisfaction served as a central mediator, reflecting its pivotal role in linking supportive work environments to improved mental health and performance outcomes [27]. Mental and physical health were integrated as significant determinants of both absenteeism and overall performance, recognizing the interplay between psychological resilience, physical capacity, and workplace engagement [28]. Absenteeism was added to capture its direct impact on performance, representing an outcome of health-related and workplace factors [29]. Lastly, sex and age were included as control variables to address potential demographic influences, including differing perceptions of workplace conditions or health outcomes [30]. However, we acknowledge that limiting the control variables to only age and sex may overlook other potentially relevant factors such as education level, job tenure, or institutional position, which could also influence the model’s outcomes. Nevertheless, this model provides a comprehensive framework for understanding and optimizing job performance in healthcare settings.

### 2.2. Research Hypothesis

The following are the alternative hypotheses of this study:

**H_1_.**  *Psychological safety affects job satisfaction.*

**H_2_.**  *Ergonomic conditions affect job satisfaction.*

**H_3_.**  *Psychological safety affects mental health.*

**H_4_.**  *Ergonomic conditions affect physical health.*

**H_5_**  : *Job satisfaction affects job performance.*

**H_6_.**  *Mental health affects job performance.*

**H_7_.**  *Physical health affects absenteeism.*

**H_8_.**  *Absenteeism affects job performance.*

**H_9_.**  *Physical health affects job performance.*

**H_10_.**  *Mental health affects absenteeism.*

### 2.3. Population and Sample

This study was conducted in Istanbul, and participants were recruited using the snowball sampling method. However, to ensure a balanced proportional distribution (50–50%) between public and private sector employees and enhance the representativeness of the sample, the method was supplemented with purposive sampling. Google Forms were used for data collection. Based on official estimates, approximately 250,000 hospital-based healthcare workers are employed in Istanbul, meaning that the sample in this study accounts for roughly 0.15% of the total population. To minimize selection bias, purposive sampling was strategically applied during the second phase to ensure institutional diversity by balancing representation between public and private hospital employees. This approach helped mitigate the overrepresentation of specific hospital types that might result from snowball sampling alone. Furthermore, the exclusion of incomplete or inconsistent responses enhanced data quality and reduced potential bias related to nonresponse patterns.

Among the participants who took part in this study as healthcare workers, 8, 1, and 25 responses were excluded owing to incomplete surveys, incorrect responses, and significant missing data in the sociodemographic questions, respectively. Overall, 79 participants were excluded from this study as they had at least one missing response to any of the scale items or did not provide an answer for the occupation variable. Consequently, the initial sample size of 452 participants decreased to 373. To compensate for the loss, additional data collection was conducted to reach 385 participants. After obtaining 12 additional valid responses, the data collection phase of this study was completed. While excluding incomplete or inconsistent responses improved data quality, we acknowledge that this process may introduce exclusion bias if specific groups (e.g., highly stressed or overburdened staff) were disproportionately represented among nonrespondents. Therefore, the final sample may underrepresent employees experiencing the highest levels of workload or distress, and this limitation should be considered when interpreting the findings.

Cochran’s sample size formula, assuming a 95% confidence level and the most conservative response distribution of 0.5, confirmed that 385 participants was sufficient. The most conservative response distribution (*p* = 0.5) was used to ensure maximum variability, which yields the largest required sample size and provides robust statistical power even in the absence of prior population estimates.

(1) $$n_{0}=\frac{1.96^{2}\times 0.5\times (1-0.5)}{0.05^{2}}=384.16\approx 385$$

Furthermore, the literature states that a minimum sample size of 385 participants is sufficient to represent a population of ≥10,000 [31]. To meet this target, this study aimed to reach 385 participants. During the second phase of data collection, the remaining 12 participants were recruited within a single day, and all of them were purposively selected from the public sector. This selection was made to maintain the proportional balance between the public and private sector participants and enhance the representativeness of the sample. As all individuals with missing responses in the scale items were excluded, some participants within the remaining 385 observations had missing responses in the sociodemographic questions. Therefore, the Missing Completely at Random (MCAR) technique was applied to determine whether these missing data followed a systematic pattern.

(2) $$\chi^{2}=\sum_{g=1}^{G}N_{g}{\left({\bar{X}}_{g}-\bar{X}\right)}^{T}S^{-1}({\bar{X}}_{g}-\bar{X})$$

The result of Little’s MCAR test showed that the *p*-value obtained from the nine missing data patterns was 0.101 (χ^2^ = 617; df = 573). As this value is >0.05, the null hypothesis could not be rejected, indicating that the missing data were completely random. Therefore, the missing values in the observations that were included in this study did not follow a systematic pattern, as they were only related to sociodemographic variables. Consequently, these observations were retained within the scope of this study.

Overall, 385 individuals participated in this study, with sociodemographic characteristics evaluated for 379–384 participants. The demographic distribution revealed that 64.64% and 35.36% of the participants were females and males, respectively. Regarding educational level, the largest group comprised bachelor’s degree holders (39.84%), followed by associate degree holders (33.07%), postgraduate degree holders (19.27%), and high school graduates (7.81%). Regarding occupation, the participants’ roles were distributed as follows: medical doctor, 55 (14.29%); nurse, 135 (35.06%); technician, 127 (32.99%); administrative staff, 36 (9.35%); psychologist, 11 (2.86%); and physiotherapist, 21 (5.45%). Regarding marital status, 42.71% of the participants were married, whereas 57.29% were single, divorced, or widowed. Regarding income level, the majority fell into the middle-income group (70.23%), with 22.72% reporting a high income and 7.05% a low income. The distribution by institution type showed that 51.05% and 48.95% were employed in public and private or foundation institutions, respectively. Participants’ ages had a range of 18–79 years, with a mean age of 37.23 ± 11.36 years (Table 1).

### 2.4. Data Collection Tools

All measurement tools employed in this study were valid and reliable scales. The SF-12 Quality of Life Scale, developed by Ware et al. [32] and adapted into Turkish by Soylu and Kütük [33], was used to assess physical health and mental health through standardized composite scores. Composite scores were calculated according to the weighted scoring procedure outlined by Ware et al., where dummy-coded item responses were multiplied by standardized coefficients and summed to generate physical and mental component scores. For instance, one item is: “*In general, would you say your health is…*” rated on a 5-point Likert scale. Higher scores in both subdimensions indicate better health.

The YS Absenteeism Scale, developed in Turkish by Yılmaz [34] includes two subdimensions, Nonmedical Absenteeism and Medical Absenteeism, calculated by averaging relevant item scores. An example item from the nonmedical dimension is: “I arrived late to work”. Responses are rated on a 5-point scale ranging from “Never” to “4 or more times”. Higher scores indicate greater absenteeism frequency.

The Job Performance (JP) Scale, developed in Turkish by Deniz and Kumru [35], consists of 10 items rated on a 5-point Likert scale. Composite scores were calculated as the arithmetic mean of all items. A sample item is: “*I pay extra attention when performing my duties*”. Higher scores reflect better job performance.

The Ergonomic Working Conditions (EWC) Scale, adapted by Oskaloğlu and Çatı [36], includes 26 items rated on a 5-point Likert scale. A sample item is: “*The temperature of my work environment is appropriate*”. Composite scores were computed by averaging item responses, with higher scores indicating more favorable ergonomic conditions.

The Job Satisfaction Scale was derived from the original 18-item scale developed by Brayfield and Rothe [37] and reduced to five items in a short form by Judge et al. [38]. Its Turkish adaptation was validated by Başol and Çömlekçi [39]. Items are rated on a 5-point Likert scale. An example item is: “*I find happiness in my job*”. Higher scores denote greater job satisfaction.

Finally, the Psychological Safety Scale, developed by Bülbül et al. [40], contains 7 items rated on a 5-point Likert scale. A representative item is: “*Employees in this workplace openly express the problems they encounter*”. Composite scores were calculated as the mean of all items, with higher values reflecting stronger perceptions of psychological safety.

### 2.5. Reliability of the Responses

The Psychological Safety Scale demonstrated high internal consistency, with a Cronbach’s alpha value of >0.80, indicating that the responses reliably measure psychological safety.

For the Job Satisfaction Scale (JS1–JS5), the Cronbach’s alpha coefficient was calculated to assess internal consistency. The analysis revealed that both the raw and standardized Cronbach’s alpha values were 0.90, indicating a high reliability level in the responses.

The composite reliability values for the physical and mental components of the SF-12 Scale, based on the participants’ responses, were 0.605 and 0.505, respectively. This result suggests that the participants’ physical health-related responses were more consistent than their responses on mental health. Notably, responses in the physical component were more evenly distributed, whereas responses in the mental component showed greater individual variation. This finding implies that participants provided more structured and homogenous responses when evaluating their physical health, whereas their mental health assessments showed greater variability.

For the YS Absenteeism Scale, excused and unexcused absenteeism-related responses were analyzed. The Nonmedical Absenteeism subdimension had a Cronbach’s alpha value of 0.61, indicating an acceptable internal consistency level. The Medical Absenteeism subdimension showed a higher reliability level, with a Cronbach’s alpha value of 0.83, indicating high consistency in the responses. When considering the Total Absenteeism score, the Cronbach’s alpha value was 0.77, confirming the overall reliability of the scale and the consistency of responses regarding absenteeism status.

The EWC Scale was analyzed in terms of its subdimensions and overall reliability. The Lighting and Accessibility subdimension exhibited a Cronbach’s alpha value of 0.81, indicating a high reliability level. The Furniture Suitability subdimension had a Cronbach’s alpha value of 0.80, whereas that of the Computer and Accessories Positioning subdimension was 0.85, both reflecting high reliability levels. However, the Work Environment Conditions subdimension had a borderline acceptable reliability level of 0.69, whereas the Assistive Equipment (0.65) and Desk Features (0.59) subdimensions demonstrated lower reliability levels. Overall, the scale demonstrated a strong internal consistency with a Cronbach’s alpha value of 0.91, confirming the reliability of the responses.

The JP Scale demonstrated a Cronbach’s alpha value of 0.92, indicating that the responses had a high internal consistency level and that participants’ responses regarding job performance were highly reliable.

### 2.6. Statistical Analysis

Data analysis was performed using R 4.4.2. Descriptive statistics included frequency, percentage, minimum, maximum, mean, and standard deviation (SD) values. Normality assumptions were evaluated on the basis of skewness and kurtosis values within the ±1.00 range. Autocorrelation was examined using the Durbin–Watson test, whereas heteroskedasticity was assessed using the Breusch–Pagan test. Multicollinearity was checked using the variance inflation factor (VIF). Depending on the distribution, either Spearman or Pearson correlation matrices were utilized. Owing to the nonnormal distribution of the dependent variable, a robust technique, the maximum likelihood robust method, was applied to mitigate the influence of outliers. In the confirmatory factor analysis (CFA), model fit was evaluated using the Chi-square/degrees of freedom ratio (χ^2^/df), comparative fit index (CFI), Tucker–Lewis index (TLI), root mean square error of approximation (RMSEA), standardized root mean square residual (SRMR), goodness-of-fit index (GFI), and adjusted goodness-of-fit index (AGFI). The linearity of the variables was assessed using scatter plots and the Ramsey RESET test; based on the results, nonlinear models were specified where appropriate. The Akaike information criterion (AIC) was used for determining the most suitable polynomial model; based on this, the degrees were identified, and the square and cube of the variable’s data were calculated accordingly. A 95% confidence level was used for statistical evaluation (Table 2).

### 2.7. Research Permits

In accordance with the Helsinki Declaration, ethical approval was obtained from the Istanbul Arel University Ethics Committee with the decision number E-52857131-050.04-730521, dated 10 January 2025, and meeting number 2025/01. The approval was officially communicated to us on 14 January 2025. The usage permissions for the scales were obtained as follows: SF-12, job performance, job satisfaction, and EWC scales were approved on 1 January 2025, whereas permissions for the psychological safety and YS absenteeism scales were obtained on 30 December 2024.

### 2.8. Inclusion and Exclusion Criteria

The following were the inclusion criteria: participants aged ≥ 18 years; healthcare workers employed in a hospital setting; fluent in Turkish; and residing in Istanbul, Türkiye during the study period. To ensure proportional representation, both public and private sector employees were included. The following were the exclusion criteria: individuals aged < 18 years; those not working in a hospital setting (e.g., administrative healthcare staff or nonhospital healthcare professionals); individuals unable to comprehend or respond in Turkish; those who did not provide informed consent; and participants with incomplete or inconsistent responses, particularly those missing at least one item from any of the study scales.

## 3. Results

Before conducting structural equation modeling, CFA was performed to assess the measurement model’s fit using the maximum likelihood robust method.

**Figure 2:**
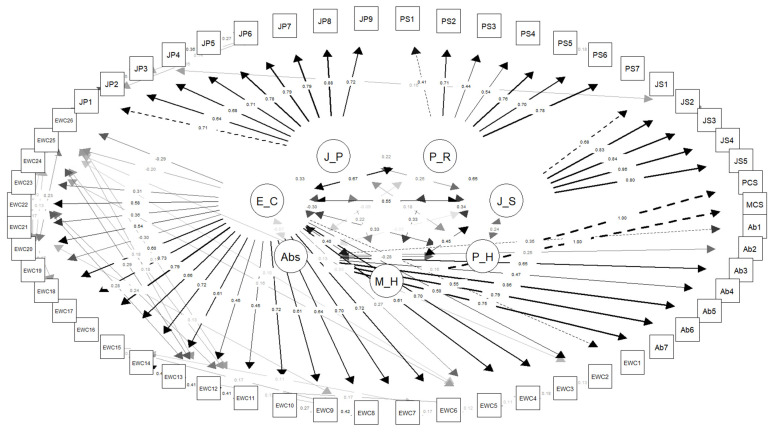
Path diagram of the confirmatory factor analysis.

The path diagram constructed to evaluate the structural equation model displays the latent variables and the factor loadings of their corresponding observed variables. In the diagram, latent variables, including psychological risks, job satisfaction, physical health, mental health, absenteeism, ergonomic conditions, and JP, are included, and these are modeled on the basis of their respective items. The fact that the vast majority of the factor loadings exceeded 0.40 indicates that the items are strongly associated with their corresponding factors. Additionally, modification indices are marked for paths that exceed a certain threshold (e.g., >6 with a positive Expected Parameter Change value), thereby identifying potential areas for improvement in the model. Precautions were taken to ensure that these modification areas occurred only among the observed variables that constituted the latent variable (Figure 2).

The results indicated an acceptable model fit based on multiple fit indices. The χ^2^/df ratio was 1.58, which is well below the threshold of 5.00, suggesting a good model fit. The CFI (0.909) and TLI (0.900), as well as their robust counterparts (0.913 and 0.905, respectively), were above the recommended 0.90 threshold, indicating a well-fitting model. Additionally, the RMSEA (0.039) and robust RMSEA (0.041) were below 0.08, further supporting a good model fit. The SRMR value (0.060) was within the acceptable range (<0.08), confirming that the model adequately represented the observed data.

The descriptive statistics for the participants’ scale scores are presented in Table 3. The psychological safety scores ranged from 7 to 35, with a mean of 23.44 (SD = 5.39), whereas the job satisfaction scores varied between 5 and 25, with an average of 18.26 (SD = 4.75). The physical health scores ranged from 19.39 to 63.83, with a mean of 46.61 (SD = 8.05), whereas the mental health scores ranged from 11.54 to 63.05, averaging 43.17 (SD = 9.77). Absenteeism had a minimum score of 1 and a maximum of 5, with a mean of 1.5 (SD = 0.6). Ergonomic conditions scores ranged from 26 to 130, with an average of 86.03 (SD = 16.08), whereas JP scores varied from 9 to 45, with a mean of 37.39 (SD = 6.46). The total sample size for the analysis was N = 385. When skewness and kurtosis values within the range of −1 to 1 were considered acceptable for normality, it was determined that the dependent variables absenteeism and JP did not follow a normal distribution. The absenteeism variable exhibited positive skewness and high kurtosis, whereas JP showed negative skewness and high kurtosis.

**Figure 3:**
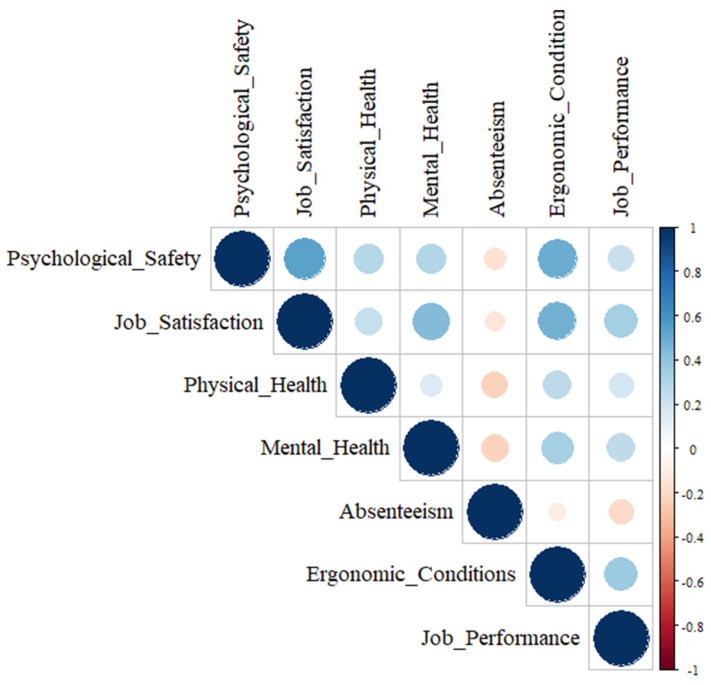
Correlation between the variables.

A strong positive correlation was noted between psychological safety and job satisfaction (r = 0.539; *p* < 0.001), indicating that a supportive and communicative work environment significantly enhances employees’ satisfaction. The strongest correlation in the matrix was between psychological safety and job satisfaction, underscoring the central role of psychological security in shaping job-related attitudes. In contrast, the weakest correlations were observed between psychological safety and absenteeism (r = −0.156; *p* = 0.002) and between job satisfaction and absenteeism (r = −0.131; *p* = 0.010), suggesting that these factors may influence absenteeism only indirectly or in interaction with other variables. Psychological safety also showed a moderate positive correlation with ergonomic conditions (r = 0.497; *p* < 0.001) and a weak positive correlation with job performance (r = 0.221; *p* < 0.001). Job satisfaction demonstrated weak-to-moderate positive correlations with physical health (r = 0.238; *p* < 0.001), mental health (r = 0.430; *p* < 0.001), ergonomic conditions (r = 0.473; *p* < 0.001), and job performance (r = 0.340; *p* < 0.001). Physical health showed only weak associations with most variables, including mental health (r = 0.160; *p* = 0.002) and job performance (r = 0.190; *p* < 0.001), and a negative correlation with absenteeism (r = −0.222; *p* < 0.001). Similarly, mental health was moderately correlated with ergonomic conditions (r = 0.334; *p* < 0.001), positively with job performance (r = 0.258; *p* < 0.001), and negatively with absenteeism (r = −0.227; *p* < 0.001). Absenteeism demonstrated consistently weak negative correlations with all other variables, including ergonomic conditions (r = −0.104; *p* = 0.041) and job performance (r = −0.196; *p* < 0.001). Finally, ergonomic conditions showed a moderate positive correlation with job performance (r = 0.364; *p* < 0.001), highlighting the role of a well-organized physical environment in boosting employee effectiveness (Figure 3).

**Figure 4:**
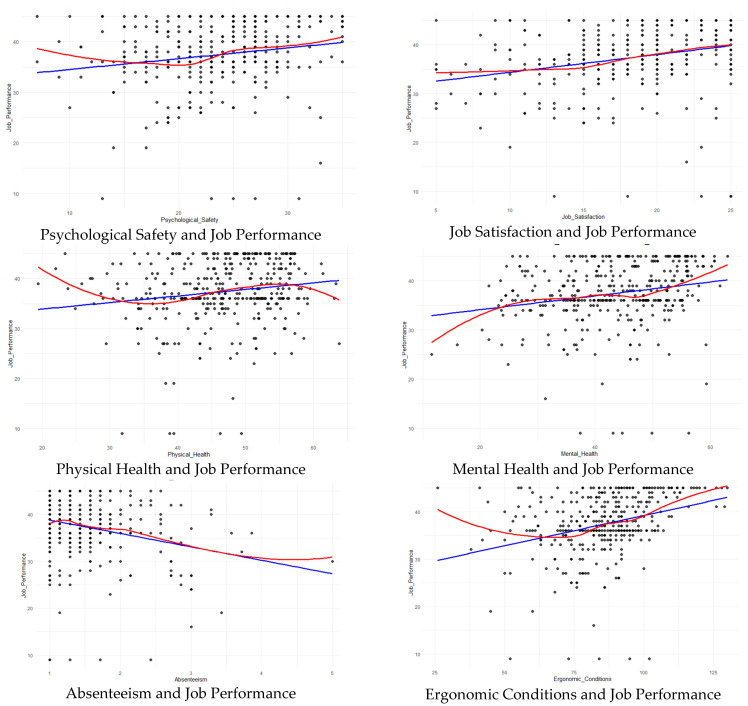
Linear and nonlinear relationships between job performance and independent variables.

In Figure 4, the linearity assumption is evaluated. A Breusch–Pagan heteroscedasticity test was performed before the linearity tests. The results of the studentized Breusch–Pagan test (BP = 9.5852, df = 6, *p* = 0.1432) indicated that the null hypothesis of homoscedasticity could not be rejected at the 95% confidence level. Thus, the model’s variance was considered homoscedastic.

Scatter plots were analyzed, and nonlinear relationships were identified. Accordingly, the Ramsey RESET test was applied to examine the linearity, and the relationships between the dependent variable (job performance) and each independent variable were analyzed with scatter plots, providing additional context. The RESET test indicated that the functional form was linear for job satisfaction (*p* = 0.4729) but nonlinear for physical health (*p* = 0.00609), mental health (*p* = 0.03592), and ergonomic conditions (*p* = 0.0005796). For psychological safety and absenteeism, the *p*-values (0.09599 and 0.1123, respectively) suggested linearity at a 95% confidence level. However, as deviations from linearity, captured by the Loess curve, were visually observed for most variables except for job satisfaction, further investigation of these nonlinear associations was performed using polynomial regression models. In the scatter plots, the blue line represents the fitted linear regression line, while the red line shows the nonlinear trend.

The following were the polynomial regression analysis results for determining the best-fitting model for each independent variable with JP: psychological safety was best modeled with a quadratic relationship (AIC = 2519.930), indicating a nonlinear relationship. Job satisfaction showed a linear relationship with JP (AIC = 2505.230). Regarding physical health, a cubic model provided the best fit (AIC = 2517.135), suggesting a more complex nonlinear relationship. Similarly, mental health was best modeled with a cubic function (AIC = 2513.149), reflecting nonlinearity. Moreover, absenteeism exhibited the best fit with a cubic model (AIC = 2505.611). Finally, ergonomic conditions demonstrated a quadratic relationship (AIC = 2481.969), indicating a nonlinear effect. Based on these results, psychological safety and ergonomic conditions were included in the structural equation model as quadratic variables, whereas physical health, mental health, and absenteeism were included as cubic variables. Job satisfaction was included with a linear relationship owing to its simplicity and best fit. Additionally, before proceeding with the structural equation modeling, multicollinearity among the independent variables was assessed using the VIF. The results indicated that all VIF values remained well below the commonly accepted threshold of 10, with the highest being 1.626 for job satisfaction. This finding suggests that the predictor variables demonstrated no problematic multicollinearity, ensuring that the estimates in the model remained stable and interpretable.

Considering the findings from the polynomial regression analysis and the absence of multicollinearity concerns, the structural equation model was constructed using the identified polynomial transformations for the respective variables.

The structural equation model (SEM) results indicated that psychological safety (std. β = 0.511, *p* < 0.001) and ergonomic conditions (std. β = 0.283, *p* < 0.001) significantly enhanced job satisfaction, with additional positive effects from sex (0: female) (std. β = 0.113, *p* = 0.009) and age (std. β = 0.091, *p* = 0.035). Job satisfaction positively influenced mental health (std. β = 0.390, *p* < 0.001), and ergonomic conditions also contributed to better mental health (std. β = 0.059, *p* = 0.039), whereas psychological safety (std. β = 0.065, *p* = 0.260), sex (std. β = −0.011, *p* = 0.824), and age (std. β = 0.165, *p* = 0.001) showed no significant impact. JP was significantly enhanced by job satisfaction (std. β = 0.225, *p* = 0.013), mental health (std. β = 0.080, *p* = 0.029), and ergonomic conditions (std. β = 0.230, *p* < 0.001), whereas absenteeism negatively affected it (std. β = −0.131, *p* = 0.012), and physical health (std. β = 0.090, *p* = 0.105), sex (std. β = 0.584, *p* = 0.410), and age (std. β = −0.020, *p* = 0.475) showed no significant effect. Absenteeism was significantly reduced by physical health (std. β = −0.157, *p* = 0.002), whereas mental health (std. β = −0.042, *p* = 0.265), sex (std. β = −0.071, *p* = 0.235), and age (std. β = 0.070, *p* = 0.067) did not demonstrate meaningful impacts (Table 4).

**Figure 5:**
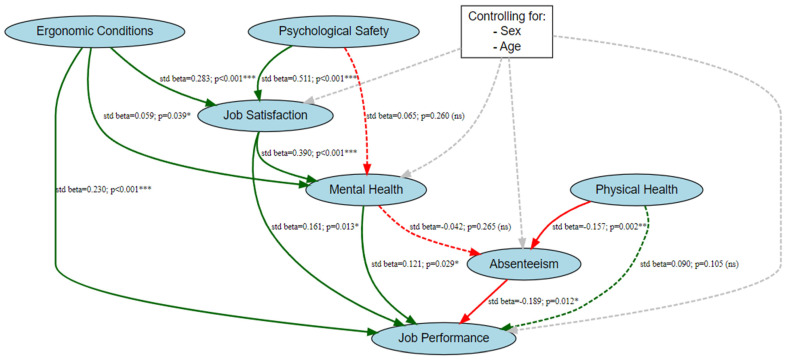
Structural equation model with pathways (*: *p* < 0.05; **: *p* < 0.01; ***: *p* < 0.001).

The structural equation model diagram underscores the intricate relationships between the variables, revealing both expected and unexpected findings. Psychological safety and ergonomic conditions significantly enhanced job satisfaction, which positively influenced mental health and JP. Contrary to the theoretical model, ergonomic conditions did not influence physical health but had a direct and significant impact on mental health and JP, emphasizing its crucial role. Physical health reduced absenteeism, and absenteeism negatively affected JP, consistent with expectations. However, psychological safety did not significantly influence mental health, and mental health showed no meaningful impact on absenteeism. Control variables (sex and age) were incorporated but did not considerably change the core relationships. The diagram visually captures these results, with significant pathways in green, nonsignificant pathways in red, and dashed lines for paths with nonsignificant effects, providing a comprehensive and nuanced representation of the model (Figure 5).

Based on multiple fit indices, the structural equation model demonstrated an excellent fit to the data. The χ^2^/df ratio was 1.08, indicating an almost perfect model fit. The CFI (1.000) and TLI (+0.999), along with their robust versions, met and exceeded the recommended ≥ 0.90 threshold, supporting an outstanding comparative model fit. The RMSEA (0.015) and SRMR (0.019) were well below the 0.08 cutoff value, signifying minimal residual error. Furthermore, the GFI (0.992) and AGFI (0.938) confirmed the strong overall model adequacy. These results indicate that the model offers an optimal data representation, closely aligning with theoretical expectations (Table 5).

## 4. Discussion

Our study, which aimed to offer valuable insights into the safety, ergonomics, and satisfaction-related dynamics of hospital work environments, showed that psychological safety and ergonomic conditions positively affected job satisfaction. This finding highlights the importance of healthcare workers in intense and stressful environments experiencing supportive and physically comfortable conditions through open communication. A study conducted on nurses reported that job demands and job satisfaction were directly related to safety performance, whereas emotional exhaustion, job resources, and job demands were all indirectly associated with safety performance [41]. Our findings suggest that the physical and psychosocial conditions of healthcare professionals in the workplace influence their satisfaction levels and shape their safety behaviors. A study investigating the satisfaction of medical laboratory employees and patient feedback observed that better ergonomic conditions improved employee satisfaction, which in turn directly positively affected patient satisfaction [42]. The direct positive effect of psychological safety and ergonomic conditions on job satisfaction observed in the present study underscores the association between employee satisfaction and patient satisfaction as well as highlights the responsibility of managers in developing ergonomic policies. A study conducted on physicians in Italy during the coronavirus disease 2019 (COVID-19) pandemic revealed that physicians’ sense of belonging to their hospitals, their belief in effective communication with patients and the organization, and their risk awareness-related nontechnical skills were positively correlated with job satisfaction. Moreover, the sense of belonging was noted to support mental well-being [43]. The manner in which employee satisfaction is shaped by communication skills with patients and the organization aligns with our finding that psychological safety and ergonomic conditions in the workplace directly affect job satisfaction. This shared perspective demonstrates that both individual factors (e.g., communication skills and sense of belonging) and environmental factors (e.g., ergonomic conditions and psychological safety) play a crucial role in enhancing job satisfaction.

The positive effect of job satisfaction on JP among healthcare workers suggests that employees are more likely to excel in workplaces where they feel appreciated, secure, and comfortable. A study conducted in Istanbul indicated that a decline in job satisfaction increased presenteeism behaviors, thereby reducing productivity and worsening JP [44]. Another study conducted among healthcare professionals during the COVID-19 pandemic revealed that JP was shaped by factors, including leadership, organizational commitment, and the work environment [45]. Similarly, a study on 527 healthcare professionals in Italy noted that psychological stress factors were associated with psychological safety and caused decreased job satisfaction [46]. A study conducted on employees with sedentary jobs in Slovenian businesses noted a positive correlation (r = 0.28 to 0.35) between work performance, well-being, job satisfaction, and life satisfaction [47]. Another study investigating innovative strategies for enhancing the sustainability of healthcare service quality highlighted that ensuring a high level of patient care quality was primarily achieved through employee job satisfaction [48]. These various studies have collectively indicated that job satisfaction directly influences not only individual well-being but also JP, patient care quality, and healthcare service sustainability. Strategies supporting psychological safety, organizational commitment, and EWC are indispensable for achieving high productivity and quality in the healthcare sector.

In our study, the expected result was also observed: physical health reduces absenteeism, and absenteeism negatively impacts JP. Although the negative relationship between absenteeism and job performance was statistically significant, the effect size was relatively modest. This suggests that other behavioral mechanisms, such as presenteeism—where employees attend work despite health issues—or quiet quitting behaviors involving minimal discretionary effort, might partially counteract the negative effects of absenteeism on performance [49]. These behaviors may maintain surface-level productivity while masking deeper engagement and well-being challenges, warranting further investigation in future studies. These findings highlight that the physical well-being of healthcare workers is crucial for job continuity and efficiency. Furthermore, recent workforce trends such as “quiet quitting”—where employees meet only the minimal requirements of their roles due to dissatisfaction or lack of psychological safety—may reflect broader disengagement processes not fully captured by absenteeism alone [49]. Our findings suggest that environments lacking in psychological safety or ergonomic support may contribute to such behaviors, subtly diminishing job performance despite physical presence. A study conducted on 2319 employees reported that employees’ physical and mental health conditions were strongly correlated with long-term absenteeism. Specifically, factors including high diastolic blood pressure, glycated hemoglobin levels, a history of heart disease, smoking, weight gain, and job stress increased absenteeism. Conversely, job suitability and the ability to utilize one’s work skills reduced absenteeism. A previous study emphasized that companies could lower absenteeism costs by investing in employees’ health [50]. A pilot study conducted among nurses reported that a resistance training program improved professional quality of life and health indicators, thereby increasing program adherence and strengthening the motivation to continue [51]. Although this finding validates that physical health reduces absenteeism and enhances JP, it also suggests that regular exercise-based workplace interventions can enhance both physical and occupational well-being, highlighting the advantages of supportive workplace elements. However, in our study, the fact that psychological safety did not show a statistically significant effect on mental health and that ergonomic conditions did not significantly affect physical health suggests that the factors determining hospital employees’ mental and physical health (e.g., long shifts, patients and workload, institutional support, and lifestyle) are multidimensional. This unexpected non-significant relationship between psychological safety and mental health may stem from several contextual factors. First, although psychological safety is conceptually associated with mental well-being, the effect may be indirect or moderated by other variables such as perceived organizational justice, workload, or peer support, which were not included in the current model. Second, given that mental health is influenced by both workplace and personal life stressors, employees might experience mental strain from sources unrelated to their immediate job environment, thus weakening the direct impact of psychological safety. Third, psychological safety in healthcare settings may contribute more to interpersonal trust and team dynamics than to individual psychological resilience. This discrepancy highlights the multidimensional nature of mental health and suggests that future models should explore mediating or moderating mechanisms between psychological safety and mental health outcomes. A study investigating the association between patient safety and nurse work–life balance demonstrated how employee stress affects JP [52]. These findings confirm that supporting employees’ physical and mental well-being is crucial for individual health, patient safety, JP, and institutional efficiency.

The nonsignificant effect of physical health on JP and the weak impact of mental health on absenteeism suggest that employees’ performance and absenteeism can be more influenced by other variables, including motivation, organizational justice, and career development, which were not investigated in this study. Nevertheless, the literature presents inconsistent findings on this matter. For example, a 2021 study on public hospital employees in Kenya revealed that intrinsic motivation played a critical role in enhancing JP [53]. Another study conducted among 395 healthcare professionals in Jimma reported that organizational justice and satisfaction with rewards increased organizational commitment and improved JP [54]. These findings imply that not only physical and mental health but also intrinsic motivation, perceptions of justice, and reward mechanisms shape employees’ JP. For sustainable job productivity and decreased absenteeism rates, healthcare institutions should develop policies that focus on physical health and strengthen employee motivation and perceptions of fairness. An experimental study on employees revealed a particularly interesting finding that under high workloads, employees exhibited job-slowing behaviors, such as presenteeism, which significantly benefited performance [55]. This observation aligns with other studies investigating the association between physical health and JP, further complicating this relationship and highlighting the differences between our theoretical model and the empirical results [56,57]. These diverse findings suggest that although the relationship between physical health and JP cannot be entirely disregarded, external factors complicate this association, introducing indirect effects that require further investigation.

The strong effect of job satisfaction on mental health and the direct impact of ergonomic conditions on both mental health and JP represent an unexpected but empirically significant finding in our theoretical model. These findings confirm that working conditions in hospitals play a critical role in shaping employees’ psychological well-being and job outcomes. A systematic review of studies conducted on nurses observed that aerobic exercise, ergonomics, and increased physical activity positively influenced work ability, likely owing to stress reduction [58]. Another literature review on healthcare professionals’ mental health examined 27 studies and reported that environmental design factors were correlated with mental health outcomes, including stress, fatigue, job satisfaction, burnout, and well-being [59]. A study conducted in India with 650 female nurses and 108 male doctors noted that nurses experienced significant occupational stress, moderate quality of life, and high musculoskeletal discomfort levels [60]. These findings suggest that ergonomic improvements and stress management strategies aimed at improving employee well-being in healthcare settings should be considered fundamental components that strengthen individual well-being, JP, and patient care quality.

Incorporating age and sex as control variables in this model originates from the widespread understanding that these demographic factors can influence employees’ attitudes and behaviors in institutional settings. In the healthcare sector, female employees tended to express significantly higher job satisfaction, which may be attributed to social roles, expectations, or responsibility distribution. A study conducted in Iran, for instance, reported that female nurses had lower job satisfaction than their male counterparts [61]. Meanwhile, age emerged as a critical factor, particularly in job satisfaction and mental health, suggesting that increasing professional experience and life knowledge can influence employees’ job perceptions and psychological resilience. A study on doctors noted that individuals aged < 45 years had higher rates of emotional exhaustion and personality disorders [62]. Therefore, controlling for basic demographic characteristics, including sex and age, enables a clearer and statistically valid assessment of institutional factors (e.g., psychological safety, ergonomic conditions, or job satisfaction). However, considering the inclusion of job roles (nurses, doctors, and physiotherapists) as a control variable, the risk of excessive dummy variables reducing statistical power was considered, resulting in the omission of this variable from the model; consequently, we acknowledged this as a limitation. Although sex and age were included as control variables in the final SEM, the exclusion of additional controls such as job title, tenure, and shift type may have limited the model’s ability to fully account for workplace-specific variation.

## 5. Conclusions

In Türkiye, public and private hospitals encounter the dual challenge of managing high patient turnover and escalating demands while also addressing significant differences in personnel management, financial resources, and organizational structure inherent to these two types of institutions. These differences necessitate individualized strategies for managing various factors. Based on our findings, psychological safety and ergonomic conditions were shown to significantly improve job satisfaction, which in turn positively influenced job performance. Moreover, physical health was found to reduce absenteeism, further supporting productivity. These empirical relationships highlight the need for targeted policies that address sector-specific workforce dynamics.

Although this study did not conduct a systematic comparative analysis between public and private hospitals, the institutional recommendations presented are informed by contextual insights and patterns frequently cited in the literature on the Turkish healthcare system. Future research could investigate whether the relationships among psychological safety, ergonomic conditions, and job performance differ significantly by hospital type. While our sample included an equal number of participants from both sectors to ensure proportional representation, organizational practices were not analyzed as moderator variables. Nevertheless, it is known that effective communication between employees and upper management may be hindered by the centralized structures and bureaucratic procedures common in public hospitals. In such settings, institutional mechanisms such as regular feedback sessions, employee engagement forums, and mediation processes can play a critical role in strengthening psychological safety. Conversely, private hospitals tend to operate in more competitive environments, which may occasionally discourage employees from voicing new ideas or taking initiative. Therefore, fostering a transparent leadership approach that supports employee confidence and strengthens organizational commitment is essential across both public and private institutions.

Typically, public hospitals operate under high patient volumes, with limited resources for improving and modernizing physical conditions, frequently constrained by long-term planning requirements. To prevent fatigue and occupational health issues among physicians and healthcare personnel, scheduled maintenance and adopting ergonomic standards should be implemented. Although private hospitals generally provide more modern equipment, cost-driven policies can occasionally lead to staff shortages in certain departments. To prevent such issues, measures including balanced shift planning, adequate equipment supply, and the provision of necessary rest areas for personnel should be employed.

Job satisfaction is essential for delivering high-quality healthcare services in both public and private healthcare sectors. As promotions and appointments in public hospitals are frequently governed by centralized regulations, continuous professional development opportunities and fair reward mechanisms should be established to enhance employee satisfaction. Although private hospitals provide attractive salary policies and rapid career development opportunities, they also require social support programs for fostering institutional commitment. Incentive mechanisms, including recognition certificates, performance-based bonuses, and flexible fringe benefits, are effective for maintaining job satisfaction in the private sector.

The high stress and burnout levels that emerge, particularly during extraordinary periods such as pandemics, natural disasters, or economic crises, pose a significant threat to the physical and mental well-being of healthcare workers. In public hospitals, the excessive patient load, combined with long working hours, can further exacerbate mental health issues, making the availability of regular counseling services and break opportunities highly beneficial. In private institutions, the emphasis on patient-centered approaches and high-performance standards can cause heightened emotional exhaustion among employees. In both settings, implementing regular health screenings, ergonomic workplace modifications, and social support initiatives can help decrease absenteeism and directly enhance JP.

The phenomenon of “quiet quitting”, where employees remain physically present at work but disengage from tasks beyond their core responsibilities, has gained prominence in recent workforce literature. This behavior often stems from low job satisfaction, diminished psychological safety, and chronic workplace stress—conditions frequently observed in both public and private hospital settings. While absenteeism is a visible indicator of workforce strain, the more subtle and pervasive impact of presenteeism and quiet quitting may reduce overall job performance without being easily captured by absence records. The findings of this study underscore the need for healthcare institutions to address not only formal attendance but also the underlying motivational and psychological factors that contribute to disengagement. By fostering psychologically safe environments and improving ergonomic conditions, institutions can mitigate the risk of quiet quitting and promote sustained engagement among healthcare workers.

Irrespective of the hospital type, addressing both physical and psychological needs within a holistic framework is significant. Strengthening both psychological safety and ergonomic standards boosts employee motivation and organizational commitment while also laying the foundation for improved service quality. In Türkiye, in particular, increasing centralized resources to alleviate excessive patient volumes in public hospitals and improve physical conditions is an urgent need. Meanwhile, in the private sector, regulatory frameworks should be established to safeguard employee rights during competitive market conditions. Consequently, a highly satisfied and skilled healthcare workforce will be nurtured, ultimately advancing the overall health standards of society.

This study, based on data from public and private hospitals in Istanbul, acknowledges its limitations while evaluating various aspects of the work environment. Specifically, restricting control variables to only age and sex has resulted in the exclusion of additional factors, including education level, years of experience, family responsibilities, and stress levels. The focus on Istanbul hospitals may also limit the generalizability of the findings to other cities or regions with different institutional structures, workforce dynamics, or healthcare policies. Moreover, our ability to assess differences among nurses, physicians, support staff, and administrative personnel has been hindered by the lack of stratification by job roles, which may further constrain the applicability of the results. The absence of direct measurements for psychosocial factors, such as burnout and perceived stress, is another limitation that may have reduced the explanatory power regarding the effects of workload-driven hospital cultures. Additionally, omitted contextual factors such as organizational culture, leadership style, and reward systems may systematically influence both risk perceptions and employee outcomes, potentially introducing unmeasured confounding into the model. Finally, as the study employed a cross-sectional design, it is inherently limited in its ability to establish causal or temporal relationships among the variables. To strengthen the understanding of these dynamics, future research should consider longitudinal or experimental designs, particularly to evaluate the lasting impact of psychological safety and ergonomic conditions on employee well-being and job performance. Despite these limitations, we believe that the simultaneous investigation of psychological safety, ergonomic conditions, job satisfaction, and performance offers valuable insights for both hospital administrators and policymakers. This study serves as a guiding resource for policymakers seeking to improve employee well-being and healthcare service quality.

## Figures and Tables

**Table 1:** Characteristics of the participants.

		n		%	
**Sex**					
Male		134		35.36	
Female		245		64.64	
*Total*		379		100	
**Educational Level**					
High School		30		7.81	
Associate Degree		127		33.07	
Bachelor’s Degree		153		39.84	
Postgraduate		74		19.27	
*Total*		384		100	
**Occupation**					
Medical Doctor		55		14.29	
Nurse		135		35.06	
Technician		127		32.99	
Administrative Staff		36		9.35	
Psychologist		11		2.86	
Physiotherapist		21		5.45	
*Total*		385		100	
**Marital Status**					
Married		164		42.71	
Single/Divorced/Widow		220		57.29	
*Total*		384		100	
**Income Level**					
Low		27		7.05	
Medium		269		70.23	
High		87		22.72	
*Total*		383		100	
**Type of Institution**					
Public		195		51.05	
Private/Foundation		187		48.95	
*Total*		382		100	
	**N**	**Min**	**Max**	**Mean**	**SD**
Age	370	17	79	37.23	11.36

**Table 2:** Confirmatory factor analysis of the model.

Fit Index and Thresholds Used	Analysis Value
χ^2^/df ≤ 5.00	1.58 (2219.55/1406)
0.90 ≤ CFI ≤ 1.00	0.909
0.90 ≤ Robust CFI ≤ 1.00	0.913
0.90 ≤ TLI ≤ 1.00	0.900
0.90 ≤ Robust TLI ≤ 1.00	0.905
RMSEA < 0.08	0.039
Robust RMSEA < 0.08	0.041
sRMR < 0.08	0.060

**Table 3:** Participants’ scores from the scales.

Variable	Min	Max	Mean	SD	Skewness	Kurtosis-3
Psychological Safety	7	35	23.44	5.39	−0.239	0.100
Job Satisfaction	5	25	18.26	4.75	−0.607	−0.049
Physical Health	19.39	63.83	46.61	8.05	−0.598	0.047
Mental Health	11.54	63.05	43.17	9.77	−0.379	−0.485
Absenteeism	1	5	1.5	0.6	1.848	4.364
Ergonomic Conditions	26	130	86.03	16.08	−0.323	0.679
Job Performance	9	45	37.39	6.46	−1.439	3.742

N = 385.

**Table 4:** Structural equation model results.

Variable	Beta	Std Error	Std Beta	z	*p*	Dependent Variable
Psychological Safety	0.010	0.001	0.511	10.908	<0.001 ***	Job Satisfaction
Ergonomic Conditions	0.000	0.000	0.283	6.018	<0.001 ***	Job Satisfaction
Sex (0: Female)	1.099	0.421	0.113	2.609	0.009 **	Job Satisfaction
Age	0.038	0.018	0.091	2.108	0.035 *	Job Satisfaction
Job Satisfaction	0.818	0.112	0.390	7.319	<0.001 ***	Mental Health
Psychological Safety	0.003	0.002	0.0655	1.126	0.260	Mental Health
Ergonomic Conditions	0.000	0.000	2.059	2.059	0.039 *	Mental Health
Sex (0: Female)	−0.217	0.976	−0.011	−0.222	0.824	Mental Health
Age	0.145	0.043	0.165	3.414	0.001 **	Mental Health
Job Satisfaction	0.225	0.090	0.225	2.488	0.013	Job Performance
Mental Health	0.080	0.037	0.080	2.190	0.029 *	Job Performance
Physical Health	0.000	0.000	0.090	1.620	0.105	Job Performance
Absenteeism	−0.131	0.052	−0.131	−2.527	0.012 *	Job Performance
Ergonomic Conditions	0.001	0.000	0.230	3.858	<0.001 ***	Job Performance
Sex (0: Female)	0.584	0.708	0.584	0.825	0.410	Job Performance
Age	−0.020	0.029	−0.020	−0.714	0.475	Job Performance
Physical Health	0.000	0.000	−0.157	−3.165	0.002 **	Absenteeism
Mental Health	−0.040	0.036	−0.042	−1.114	0.265	Absenteeism
Sex (0: Female)	−1.389	1.170	−0.071	−1.187	0.235	Absenteeism
Age	0.059	0.033	0.070	1.829	0.067	Absenteeism

*: *p* < 0.05; **: *p* < 0.01;***: *p* < 0.001

**Table 5:** SEM model goodness-of-fit indices.

Fit Index and Thresholds Used	Analysis Value
χ^2^/df ≤ 5.00	1.08 (6.508/6)
0.90 ≤ CFI ≤ 1.00	1.000
0.90 ≤ Robust CFI ≤ 1.00	1.000
0.90 ≤ TLI ≤ 1.00	0.999
0.90 ≤ Robust TLI ≤ 1.00	0.999
RMSEA < 0.08	0.015
Robust RMSEA < 0.08	0.015
SRMR < 0.08	0.019
0.85 ≤ GFI ≤ 1.00	0.992
0.85 ≤ AGFI ≤ 1.00	0.938

## Data Availability

The original data presented in this study are openly available at https://zenodo.org/records/15280777, (accessed on 25 April 2025).
